# Percutaneous cervical cordotomy for cancer-related pain: national data

**DOI:** 10.1136/bmjspcare-2019-002057

**Published:** 2020-03-27

**Authors:** Marlise Poolman, Matthew Makin, Jess Briggs, Kate Scofield, Nick Campkin, Michael Williams, Manohar Lal Sharma, Barry Laird, Catriona R Mayland, INPIC Group, John Ellershaw

**Affiliations:** 1 Bangor Institute for Health and Medical Research, Bangor University, Bangor, Gwynedd, UK; 2 Pennine Acute Hospitals NHS Trust, Manchester, UK; 3 The Christie NHS Foundation Trust, Manchester, UK; 4 St Columba's Hospice, Edinburgh, UK; 5 Queen Alexander Hospital, Portsmouth Hospitals NHS Trust, Portsmouth, UK; 6 Department of Pain Medicine, Walton Centre NHS Foundation Trust, Liverpool, UK; 7 Institute of Genetics and Molecular Medicine, The University of Edinburgh, Edinburgh, UK; 8 Department of Oncology and Metabolism, The University of Sheffield, Sheffield, UK; 9 Palliative Care Institute, University of Liverpool, Liverpool, Merseyside, UK

**Keywords:** cancer, clinical decisions, pain

## Abstract

**Objectives:**

Percutaneous cervical cordotomy (PCC) is an interventional ablative procedure in the armamentarium for cancer pain treatment, but there is limited evidence to support its use. This study aimed to assess the effectiveness and safety of PCC.

**Methods:**

Analysis was undertaken of the first national (UK) prospective data repository of adult patients with cancer undergoing PCC for pain treatment. The relationship between pain and other outcomes before and after PCC was examined using appropriate statistical methods.

**Results:**

Data on 159 patients’ PCCs (performed from 1 January 2012 to 6 June 2017 in three centres) were assessed: median (IQR) age was 66 (58–71) years, 47 (30%) were female. Mesothelioma was the most common primary malignancy (57%). The median (IQR) time from cancer diagnosis to PCC assessment was 13.3 (6.2–23.2) months; PCC to follow-up was 9 (8–25) days; and survival after PCC was 1.3 (0.6–2.8) months. The mean (SD) for ‘average pain’ using a numerical rating scale was 6 (2) before PCC and 2 (2) at follow-up, and for ‘worst pain’ 9 (1) and 3 (3), respectively. The median (IQR) reduction in strong opioid dose at follow-up was 50% (34–50). With the exception of ‘activity’, all health-related quality of life scores (5-level version of EuroQol-5 Dimension) either improved or were stable after PCC. Six patients (4%) had PCC-related adverse events.

**Conclusions:**

PCC is an effective treatment for cancer pain; however, findings in this study suggest PCC referrals tended to be late in patients’ disease trajectories. Further study into earlier treatment and seeking international consensus on PCC outcomes will further enhance opportunities to improve patient care.

## Introduction

Pain control reduces suffering and improves quality of life. The WHO analgesic ladder serves as a foundation for the treatment of cancer-related pain.^1^ However, in 10%–20% of patients, standard therapies (eg, opioids) are not effective and other strategies are needed.^2^ In patients with uncontrolled cancer pain, interventional techniques, such as percutaneous cervical cordotomy (PCC), should be considered.^3^

PCC is a procedure carried out in a conscious patient and uses radiofrequency ablation to create a heat lesion in the lateral spinothalamic tract in the upper cervical spinal cord, interrupting ascending pain signals.^4^ A successful procedure results in an analgesic area below the C4 dermatome on the contralateral side of the body, and therefore may have particular utility in pain syndromes that are predominantly one sided (eg, malignant pleural mesothelioma). The goal is to achieve diminished pinprick sensation and loss of temperature perception in the painful region of the body. This can be done as an open surgical exposure but most commonly is through a percutaneous route—PCC.

Cordotomy is indicated for unilateral cancer pain in patients with a life expectancy of less than 1 year who are not responding to other interventions.^5^ Beyond this time period, some patients can develop neuropathic deafferentation pain as a postprocedure complication; and as such PCC is only indicated in patients with limited life expectancy.^5^

Despite PCC being an important part of the armamentarium for the treatment of cancer pain, there is limited evidence to support its use. A systematic review by our group, focused on the effectiveness of PCC in mesothelioma-related pain,^6^ found the available evidence is significantly limited in quantity and quality. There is a lack of clear consensus as to whether PCC is most effective for neuropathic, nociceptive or indeed mixed pain.^7 8^ One of the advantages of PCC is that mobility is preserved (eg, compared with other interventions such as neuraxial analgesia that are often an alternative option in such cases).^9^

Although the research done suggests that PCC might be safe and effective, more work is needed to inform clinical practice and support its continued provision.^6^ There is a need for a prospective evaluation of the efficacy and complications of PCC for cancer pain. Our research group established a national (UK) PCC register in 2012. In this register, we prospectively collect key data on all patients who have PCC performed for cancer-related pain, both before procedure and at follow-up. Herein we present data from the first cohort of patients from the register.

## Methods

Following a UK-wide survey and subsequent Delphi process^10^ focused on the role of PCC in the management of mesothelioma, a national PCC data set was finalised by the project team. A medical database information technology company Dendrite Clinical Systems (www.e-dendrite.com) was requisitioned to host the repository.

The registry was launched in January 2012, which prospectively collects key preprocedure and follow-up data on all patients who have PCC for cancer pain. To our knowledge, this is the first national data repository for PCC in the world.

Data from this PCC repository register were assessed between 1 January 2012 and 6 June 2017. The register collected data from three interventional pain centres offering cordotomy procedures (Liverpool, Portsmouth and Warwick) serving a geographically defined population of 60 million. A further centre in Oldham was not included within this analysis due to limited resources to input data and its subsequent closure in 2015.

### Patients

All patients in the register met the following criteria: pathological or radiological diagnosis of cancer; pain secondary to their cancer (≥4/10 (worst pain in the previous 24 hours) on a 0–10 numerical rating scale (NRS)); PCC indicated following PCC practitioner assessment; and over 18 years of age. Additionally, the PCC practitioner used their clinical judgement to ensure each patient had underwent a reasonable trial of opioids and adjuvant medication.

### PCC procedures

The PCC was performed as an inpatient, under fluoroscopic guidance (X-ray) and performed by one of five pain medicine consultants trained in this technique^11^ and regularly undertaking this procedure (minimum five procedures per year). The equipment used (needles and C-arm for imaging) were from each centre’s usual suppliers, and each centre has a bespoke head support.

Typically, patients required sedation for the procedure using either fentanyl alone, or in combination with sedatives (eg, propofol). Patients were placed in the supine position with their head and neck stabilised in an external fixator (head support). An image intensifier and contrast (after dural puncture) were used to identify C1 and C2 vertebrae and the dentate ligament on the side contralateral to the pain. A radiofrequency electrode was passed into the anterolateral spinal cord and the spinothalamic tract then localised through sensory stimulation. Incremental heat lesions were created in the lateral spinothalamic tract while assessment was made of ipsilateral motor function to ensure preservation of the adjacent corticospinal tract. The contralateral analgesia was then checked by pinprick and the power of the ipsilateral arm and leg was also checked between each lesion. If required the procedure was repeated. The total PCC procedure time was usually under 60 min, most lasting 30–45 min. The policy in all study centres was that strong opioid analgesics were reduced by up to 50% of total daily dose either immediately before or after procedure.

### Assessments

The following were assessed before and after PCC: pain (average and worst) using a 0–10 NRS; health-related quality of life using the 5-level version of EuroQol-5 Dimension (EQ-5D-5L) (where a lower score indicates a less negative impact on quality of life and function, and dimensions include mobility, self-care, usual activities, pain/discomfort, anxiety/depression)^12^; and strong opioid medication (morphine equivalent daily dose (MEDD) was calculated to allow comparison). Side effects/adverse events were assessed descriptively following each procedure and recorded in case records. Further presence of specific side effects was assessed including urinary retention, opioid toxicity, dyspnoea and postprocedure pain at PCC site. Data were collected and entered into the registry either by the pain specialist nurse or the PCC practitioner.

### Analysis

Primarily, a descriptive analysis was undertaken. The primary purpose of the repository was data collection so formal hypothesis testing and sample size calculation were not appropriate. However, exploratory analyses were undertaken using appropriate non-parametric tests. Data checking (to ensure consistency with care records) was conducted. Only those patients who had data collected as part of the repository were assessed. All statistical analyses were performed using SPSS V.21.0. Where appropriate, means and SDs, or medians and IQR are reported throughout. A p value <0.05 was regarded as statistically significant.

## Results

From 1 January 2012 to 6 June 2017, one hundred and fifty-nine patients had a PCC performed and were analysed as part of the present study. Patient demographics and baseline pain characteristics are shown in table 1.

**Table 1:** Patient demographics and baseline pain characteristics (n=159)

	n (%)
Female	47 (30)
Place of care (when patient was referred for PCC)	
Home	109 (69)
Hospice	39 (25)
Hospital	10 (6)
Other	1 (1)
Diagnosis	
Malignant pleural mesothelioma	90 (57)
Primary lung cancer	43 (27)
Other cancer	26 (17)
Performance status (ECOG)	
1	36 (23)
2	61 (38)
3	59 (35)
4	3 (2)
Source of referral for PCC	
Specialist palliative care	96 (60)
Oncology	31 (20)
Respiratory medicine	14 (9)
Pain medicine	13 (8)
General practitioner	3 (2)
Other	1 (1)
Primary site of pain	
Thorax	136 (86)
Arm	17 (11)
Leg	6 (4)

ECOG, Eastern Cooperative Oncology Group; PCC, percutaneous cervical cordotomy.

The median (IQR) age of patients was 66 (58–71) years. All but two patients (n=157) were on strong opioid analgesia (eg, morphine). The median (IQR) strong opioid dose (MEDD) at baseline was 200 mg (100–400). The median (IQR) time from diagnosis of cancer to assessment for consideration of PCC was 13.3 (6.2–23.2) months. One hundred and twenty-nine patients (81%) had their cordotomy done within 48 hours of assessment by their PCC practitioner. The median (IQR) time to follow-up was 9 (8–25) days and within this time period 137 (87%) patients were alive. The median (IQR) duration of survival from procedure was 1.3 (0.6–2.8) months.

**Figure 1:**
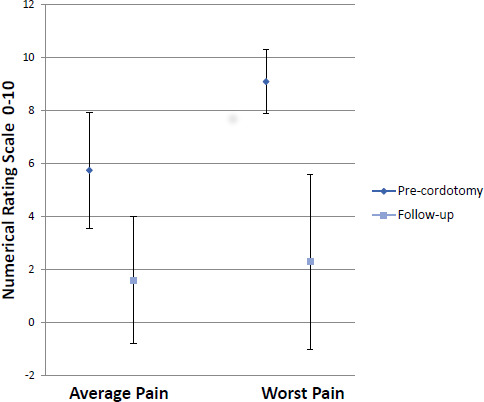
Average and worst pain, before procedure and at follow-up (n=159).

Figure 1 shows the average and worst pain (using a 0–10 NRS), before PCC and at follow-up. The mean (SD) for average pain was 6 (2) before cordotomy and 2 (2) at follow-up, p<0.001 (Wilcoxon signed-rank test). The mean (SD) for worst pain was 9 (1) before cordotomy and 3 (3) at follow-up, p<0.001 (Wilcoxon signed-rank test). The median (IQR) reduction in strong opioid dose at follow-up was 50% (34–50). (At baseline, median (IQR) MEDD=200 mg (100–400 mg), at follow-up=140 mg (20–300 mg), p<0.001 (Kolmogorov-Smirnov test).)

**Figure 2:**
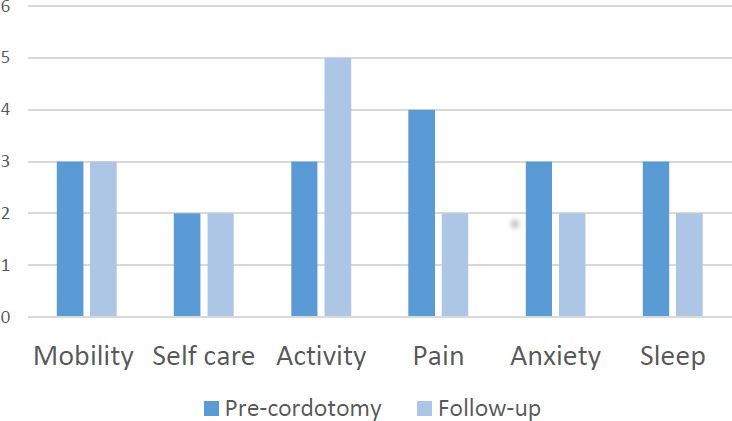
5-Level version of EuroQol-5 Dimension (EQ-5D-5L), before procedure and at follow-up (n=110).

Figure 2 shows the EQ-5D scores before cordotomy and at follow-up. There was an improvement in activity after procedure (p=0.018) but a reduction in sleep (p<0.01)—Wilcoxon signed-rank test. In other areas, all patient scores either improved or were stable after cordotomy.

Six patients (4%) had adverse events relating to their PCC including urinary retention (2) opioid toxicity (1) impaired balance (1) dyspnoea (1) and pain at the PCC site (1).

## Discussion

### Statement of main findings

The findings from our data repository suggest PCC is an effective treatment for patients with cancer-related pain and helps facilitate a significant reduction in opioid medication. The findings support previous observations that cordotomy has an ongoing role in the management of unilateral pain due to malignancy such as mesothelioma and lung cancer.^6^ Of equal importance is that in terms of PCC’s effectiveness for improving pain control, its benefits far outweigh its risks as shown by the fact that only six patients (4%) reported any adverse effects. This aligns with conclusions in previous work noting that serious complications related directly to the procedure are minimal providing the procedure is done by an experienced clinician.^5 6^ Additionally, the overall quality of life for patients undergoing PCC improved or was maintained at the same level.

It was also observed that patients tended to receive the procedure late in their disease trajectory (averaging just over a month before their death) and so potentially did not receive prolonged benefit from this intervention. Although this may relate to pain control issues not being significantly severe to merit a PCC, the services as a whole advocate early review to optimise procedure timing. It was noteworthy that 20 patients died after PCC and before the initial follow-up. This observation likely represents real-life clinical practice where factors such as overestimation of prognosis, uncertain disease trajectory and referrals being made too late are occurring. We also hypothesise that the lack of familiarity and limited access to PCC services may have compounded the aforementioned factors. Given the limited survival of these patients, getting the timing right for PCC is crucially important.

### How this relates to the existing literature

A previous review article summarised six case series, totalling 677 patients with cancer, who were treated with unilateral cordotomy since 1990.^13^ Similar benefits to our study in terms of pain control and opioid consumption were reported. A further two case series (with 41 and 207 participants) have also shown benefits in terms of pain relief for PCC using CT.^9 14^

Within this study, the most common source of PCC referral came from specialist palliative care teams. This may reflect the complex nature of the pain, and hence the need for specialist input. Alternatively, it may reflect a lack of awareness about the availability of the procedure. Within the UK, it is important to recognise that the number of centres offering PCC has reduced in recent years. One of the centres, in Warwick, closed in 2015 due to practitioner retirement; a further centre in Oldham, not included within this analysis due to limited resources to input data, also closed in 2015. Currently, there are only three centres (Portsmouth, Liverpool and, since 2017, Glasgow) which offer the procedure, with less than 10 practitioners nationally who are trained to perform the procedure. This impacts patients, who may already have significant morbidity from their illness, who then have to travel substantial distances to be considered for a PCC. Internationally, this decline is also recognised with cordotomy being considered a ‘dying art’ within North America and the practice of PCC being infrequently used.^15^ Within the last decade, only three medical centres within the USA have reported on their PCC performance.^16^ Globally, unless PCC is highlighted as an essential procedure for the management of complex pain, and specific teaching provided within specialist training programmes, expertise will disappear.

### Strengths and limitations

This is the first national PCC data repository and one of the largest within the last 20 years.^17^ Although a larger case series (n=207) has been published,^9^ this was over a 20-year period, and it was uncertain if the data represented national findings. Additionally, we have demonstrated the potential strength of this data repository—recording standardised patient-related outcome measures to establish the effectiveness and safety of a specific procedure.

The study has several weaknesses. First, the numbers are modest as over a 5-year time period, 159 procedures were conducted. Second, the study is observational in nature, so lacks a comparison or control group to be able to draw more definitive conclusions about the implications for clinical practice. For example, the majority of patients had pain in the thoracic region and pain appeared to respond well to PCC. The lack of a sufficiently sized comparator group (eg, upper limb pain) meant that comparison in efficacy dependent on location was not possible, but this would be of interest in future work. Third, although pain was assessed, other outcomes (eg, effect of physical activity) would also be of interest. Additionally, pain characterisation was limited; specifically whether pain was predominantly nociceptive or neuropathic was not consistently recorded. As previous work in this area would suggest that some pain types may respond better to PCC, garnering this in future work would be of importance. Finally, the degree of missing data limits the level of information provided such as specific details about the pain quality, more robust data on non-opioid analgesics and the provision of non-pharmacological strategies to manage the pain. More robust and systematic collection of data in these areas would be beneficial.

### Implications and unanswered questions

Further recognition about the role of PCC within the patients’ trajectory would seem pertinent. With patients dying a mean of 1.3 months following PCC, potentially patients were not always deriving full benefit from the procedure. Additionally, some patients died following their initial assessment suggesting that there may have been an overestimation of prognosis. Hence, one of the key issues relates to the definition of clear triggers for considering PCC in a timely manner. Generally, PCC tends to be considered for those with unilateral pain (especially when secondary to costopleural syndrome), who are experiencing inadequate pain control, have dose-limiting adverse effects or a combination of both of these factors.^5^ Alternatively, indication for PCC is defined as ‘medically refractory, unilateral pain in a patient with an expected lifespan of less than one year’.^15^ Patients in the present cohort were not referred for cordotomy based on any formal criteria. Rather referral was made considering multiple factors including patients’ condition (function and prognosis), analgesic therapy, patient and family preference and ease of travel to the treating centre. Future work which highlighted patients most likely to benefit from cordotomy would be of interest.

Although centres were able to offer the procedure quickly (81% had PCC within 48 hours of assessment by a PCC practitioner) and respond well to patients’ needs, earlier referral for PCC could have provided better quality of life for a longer time period. Previous consensus work agreed that PCC should be considered when the patient is requiring strong opioids and/or symptoms persist or escalate following systematic oncology interventions.^5^ All but two patients within this data repository were receiving opioid analgesia. This raises the question as to whether this is sufficiently discriminative to aid timely referral. Clearer ‘trigger points’ for patient identification are required to ensure maximum patient benefit from PCC and represent an area for further clarity.

The present findings support the role of PCC yet the limitations highlight the need for future work which should include a comparator group, detailed characterisation of those referred including non-opioid and non-pharmacological strategies, geographical site and adverse events.

Additionally, accepting that there may be a limited number of centres offering PCC, assessing innovative methods for ‘remote consultations’ would be a key line of exploration. Finally, seeking wider international consensus on routinely recording specific patient-related outcome measures for PCC would enhance numbers and therefore the robustness of recommendations.

## Conclusion

The present findings support the role of PCC as an effective and safe treatment for cancer-related pain. We propose that patients who have pain which may be adequately treated by PCC should be considered for this at the earliest opportunity, rather than progressing through multiple types of analgesia which may be suboptimal but also have side effects related to these. Patients were often referred late in their disease and potentially the full benefit of this procedure was not realised; it is not clear if this observation was specific just to the UK or reflects wider practice. Further work is needed to fully elucidate this and to assess potential wider benefits of PCC.

### Research reporting checklists

As this paper reports the results of an observational study using routinely collected health data, we used the Strengthening the Reporting of Observational Studies in Epidemiology extension RECORD (REporting of studies Conducted using Observational Routinely collected health Data) checklist.
